# Phylogeography and Conservation Genetics of the Common Wall Lizard, *Podarcis muralis*, on Islands at Its Northern Range

**DOI:** 10.1371/journal.pone.0117113

**Published:** 2015-02-06

**Authors:** Sozos Michaelides, Nina Cornish, Richard Griffiths, Jim Groombridge, Natalia Zajac, Graham J. Walters, Fabien Aubret, Geoffrey M. While, Tobias Uller

**Affiliations:** 1 Edward Grey Institute, Department of Zoology, University of Oxford, OX1 3PS, Oxford, United Kingdom; 2 States of Jersey, Department of the Environment, Howard Davis Farm, La Route de la Trinite, Trinity, Jersey, JE3 5JP, Channel Islands, United Kingdom; 3 Durrell Institute of Conservation and Ecology (DICE), School of Anthropology and Conservation, University of Kent, Canterbury, Kent, CT2 7NR, United Kingdom; 4 International Institute for Culture, Tourism and Development, London Metropolitan University, 277–281, Holloway Road, London, N7 8HN, United Kingdom; 5 Station d’Ecologie Expérimentale de Moulis, CNRS, 09200, Saint-Girons, France; 6 School of Biological Sciences, University of Tasmania, PO Box 55, Hobart, Tas, 7001, Australia; 7 Department of Biology, Lund University, Sölvegatan 37, SE 223 62, Lund, Sweden; Smithsonian Conservation Biology Institute, UNITED STATES

## Abstract

Populations at range limits are often characterized by lower genetic diversity, increased genetic isolation and differentiation relative to populations at the core of geographical ranges. Furthermore, it is increasingly recognized that populations situated at range limits might be the result of human introductions rather than natural dispersal. It is therefore important to document the origin and genetic diversity of marginal populations to establish conservation priorities. In this study, we investigate the phylogeography and genetic structure of peripheral populations of the common European wall lizard, *Podarcis muralis*, on Jersey (Channel Islands, UK) and in the Chausey archipelago. We sequenced a fragment of the mitochondrial cytochrome b gene in 200 individuals of *P. muralis* to infer the phylogeography of the island populations using Bayesian approaches. We also genotyped 484 individuals from 21 populations at 10 polymorphic microsatellite loci to evaluate the genetic structure and diversity of island and mainland (Western France) populations. We detected four unique haplotypes in the island populations that formed a sub-clade within the Western France clade. There was a significant reduction in genetic diversity (H_O_, H_E_ and A_R_) of the island populations in relation to the mainland. The small fragmented island populations at the northern range margin of the common wall lizard distribution are most likely native, with genetic differentiation reflecting isolation following sea level increase approximately 7000 BP. Genetic diversity is lower on islands than in marginal populations on the mainland, potentially as a result of early founder effects or long-term isolation. The combination of restriction to specific localities and an inability to expand their range into adjacent suitable locations might make the island populations more vulnerable to extinction.

## Introduction

There is a growing interest in the patterns and processes associated with geographical variation in population genetic structure across species’ ranges since these often shift, expand and contract over time [1–4]. Historical and contemporary changes to population size and gene flow influence genetic diversity and population differentiation [3,5]. These changes are particularly important in populations at geographical range limits, since these populations experience more rapid cycles of extinction, recolonization (with the associated founder events), severe population bottlenecks and asymmetric gene flow [3]. As a consequence, marginal populations tend to show greater than expected isolation by distance and have lower genetic diversity than populations located within the species’ range [3]. They are therefore often of particular conservation interest [6,7].

To complicate matters, it is increasingly recognized that isolated populations at the edge of species’ distributions might not have dispersed, or become isolated, naturally but instead might have been assisted by humans. This has the potential to result in genetic admixture when animals are introduced from multiple source populations. As a consequence of human-mediated dispersal and resulting admixture, marginal populations might actually show higher genetic diversity than geographically more central populations [8,9]. Therefore, it is important to establish the origin of marginal populations to be able to assign conservation priorities. This is well exemplified by the changing status of the pool frog (*Pelophylax lessonae*) in Britain. Initially considered to be present solely as a result of human introductions the native status of pool frogs was confirmed just in time to witness its extinction [10]. The species is now the focus of an active reintroduction program [11].

The common wall lizard (*Podarcis muralis*) exhibits a wide distribution across central and southern Europe. It also occurs in peripheral populations in Northern Europe where its status as a native species is debated. For example, while populations of wall lizards are known to be non-native in England [12] and parts of Germany [13], some isolated populations at the northern range limit in France, the Netherlands, and in Eastern Europe are of uncertain origin [14]. Of particular interest are populations on islands in the Golfe Normand-Breton, which were previously part of the French continental landmass and have been separated following climate and sea level changes about 7,000 BP [15,16]. Jersey, the largest of Channel Islands (11,630ha) [17] and the Chausey archipelago (a group of islands, totaling 59ha) are now 25.5 and 17 km west of Normandy Coast, respectively [17,18]. The presence and distribution of wall lizards on Jersey has been described by a number of authors [19–21] and it has been widely assumed that *P*. *muralis* is native to these islands. However, the species distribution on Jersey is noticeably patchy and restricted to old walls and ramparts on the north-eastern and eastern coast of the island [22], which suggests that they could have been introduced following the construction of the forts. Indeed, a population on the south east coastline of Jersey, cut off from the rest of the Island at high tide, is known to be a more recent introduction, although the origin of those animals is unknown [23].

The origin and genetic diversity of populations of *P*. *muralis* on the Channel Islands is of much interest as they are currently considered threatened and enjoy full protection status, despite that its present distribution is indicative of more recent introductions. Natural colonization of islands could have occurred from southern refugia, following climatic warming at the end of the Pleistocene and before the rising sea level, followed by separation from the mainland. Alternatively, colonization could have occurred subsequent to island isolation via rafting or the quarrying of granite. The aim of this study was to infer the origin of *P*. *muralis* populations on Jersey and Chausey Island and investigate the population genetic structure and diversity in relation to mainland populations. Based on our results we discuss conservation implications for these peripheral populations.

## Materials and Methods

### Study species

The European wall lizard, *Podarcis muralis* (Laurenti, 1768) has a wide distribution in central and southern Europe [24] and shows a strong phylogeographic structure with several genetically and geographically distinct clades [25,26]. This genetic structure is likely to have originated during isolation in southern glacial refugia in Italy on the Apennine Peninsula [25], the Balkans and on the Iberian Peninsula [24,26]. The postglacial recolonization of western Europe expands to the northwest along the French coast of the English Channel, across southern Belgium and southernmost Netherlands towards south-western Germany [24].

### Sampling, sequencing and genotyping

**Fig 1:**
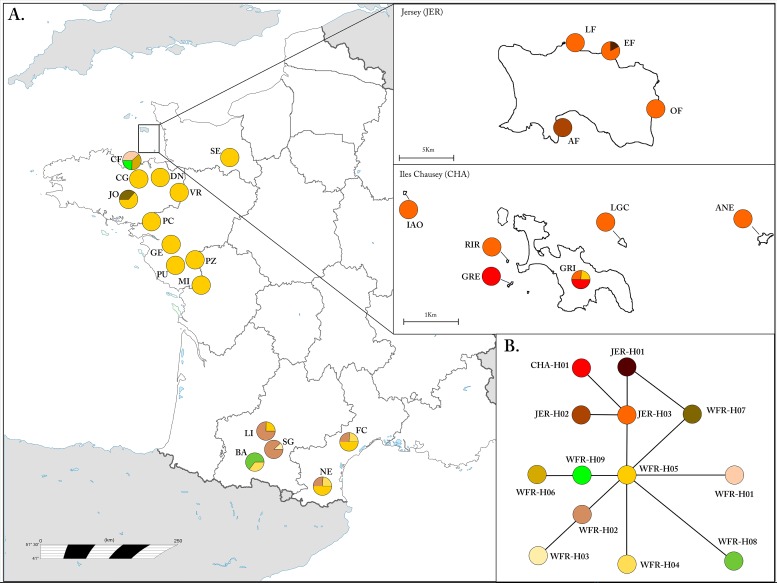
Distribution of sampled sites and haplotype network. (A) Pie charts indicate the percentage of sampled individuals matched to a specific haplotype (for population abbreviations see Table 1). (B) Parsimonious phylogenetic network reconstructed from 13 unique haplotypes sampled in our populations using a median-joining algorithm.

We sampled 484 individuals from 21 populations between 2008 and 2013 (see Table 1 and Fig. 1 in results section). We sampled lizards from all four locations on Jersey (St. Aubin Fort, Mont Orgueil Castle and Gorey, L’Etacquerel Fort and Fort Leicester, see Table C in S1 File for more information), from the Chausey archipelago (where the lizard is more widespread, see Table C in S1 File for more information) and from 19 populations in France (see Table C in S1 File for more information). We focused on mainland populations at the northwestern margin of the species distribution, i.e., close to the Channel Islands, but also included a number of populations in south-western France to compare the observed divergence between island populations with divergence across the entire western France lineage.

**Table 1:** Results from mtDNA and microsatellite analyses.

Region	Population	Code	Latitude (^o^N)	Longitude(^o^E)	N_I_ *	N_H_	Haplotype**	A_R_	H_O_ (s.d)	H_E_ (s.d)	F_IS_ _***_
Jersey	St. Aubin Fort	AF	49.18712	-2.17103	15(15)	1	JER-H2(15)	2.12	0.237	0.409	**0.452**
									(0.071)	(0.063)	
	L’Etacquerel Fort	EF	49.238267	-2.06698	17(17)	2	JER-H1(2)	2.14	0.313	0.401	**0.255**
							JER-H3(15)		(0.077)	(0.075)	
	Fort Leicester	LF	49.240243	-2.08162	14(14)	1	JER-H3(14)	2.86	0.375	0.532	**0.35**
									(0.104)	(0.079)	
	Mount Orgueil Castle	OF	49.198904	-2.02013	34(34)	1	JER-H3(35)	2.71	0.403	0.552	**0.291**
									(0.1)	(0.082)	
Chausey Archipelago	Iles de Chausey	CH	48.87425	-1.83016	31(34)	3	JER-H3(30)	3.21	0.547	0.613	0.144
							CHA-H1(3)		(0.104)	(0.084)	
							WFR-H5(1)				
France	Cap Frehel	CF	48.66451	-2.32066	12(11)	3	WFR-H1(6)	2.92	0.508	0.558	0.134
							WFR-H6(3)		(0.115)	(0.101)	
							WFR-H9(2)				
	Chateau du Guildo	CG	48.574464	-2.20691	25(5)	1	WFR-H5(5)	3.05	0.528	0.609	**0.155**
									(0.092)	(0.091)	
	Dinan	DN	48.454352	-2.04734	25(5)	1	WFR-H5(5)	3.35	0.630	0.646	0.045
									(0.049)	(0.046)	
	Sees	SE	48.605425	0.172979	24(5)	1	WFR-H5(5)	2.46	0.451	0.480	0.085
									(0.078)	(0.078)	
	Vitre	VR	48.124012	-1.2144	20(5)	1	WFR-H5(5)	3.52	0.590	0.632	0.092
									(0.079)	(0.081)	
	Josselin	JO	47.953899	-2.54648	25(5)	2	WFR-H5(3)	3.63	0.589	0.634	0.091
							WFR-H7(2)		(0.086)	(0.083)	
	Pontchateau	PC	47.436895	-2.08903	25(5)	1	WFR-H5(5)	3.76	0.513 (0.091)	0.550 (0.093)	0.088
	Puybelliard	PU	46.706436	-1.02946	22(5)	1	WFR-H5(5)	3.61	0.662	0.699	0.079
									(0.079)	(0.081)	
	Pouzagues	PZ	46.78435	-0.83917	25(5)	1	WFR-H5(5)	3.80	0.694	0.718	0.054
									(0.076)	(0.080)	
	Saint Gervais	GE	46.902738	-1.99874	25(5)	1	WFR-H5(5)	3.66	0.659	0.706	0.088
									(0.085)	(0.084)	
	Bastide	BA	42.939334	1.055994	25(5)	2	WFR-H3(2)	3.26	0.564	0.635	0.041
							WFR-H8(3)		(0.109)	(0.095)	
	Saint Michel	MI	46.353210	-1.25172	25(5)	1	WFR-H5(5)	3.56	0.686	0.699	0.151
									(0.086)	(0.082)	
	Saint Lizier	LI	43.003259	1.138791	20(5)	2	WFR-H2(4)	3.8	0.704	0.708	0.031
							WFR-H5(1)		(0.085)	(0.087)	
	Saint Girons	SG	42.982243	1.146273	25(5)	2	WFR-H2(4)	3.66	0.639	0.707	**0.12**
							WFR-H3(1)		(0.108)	(0.074)	
	Nebias	NE	42.896786	2.11586	25(5)	3	WFR-H5(3)	3.77	0.625	0.718	**0.15**
							WFR-H2(1)		(0.079)	(0.079)	
							WFR-H4(1)				
	Fontiers Cabardes	FC	43.369587	2.248493	25(5)	3	WFR-H4(1)	3.45	0.610	0.650	0.087
							WFR-H5(3)		(0.105)	(0.098)	
							WFR-H2(1)				

* Number of individuals used in microsatellite analysis and in parenthesis the number of individuals used in mtDNA analysis.

** Number of individuals sharing the same haplotype is shown in parenthesis

*** Values in bold indicate significant deviation from Hardy-Weinberg equilibrium after correcting for multiple tests at the nominal level (5%), *p>0*.*00024*.

N_I_ (number of individuals), N_H_ (number of haplotypes), A_R_ (allelic richness), H_O_ (observed heterozygosity), H_E_ (expected heterozygosity) and F_IS_ (inbreeding coefficient).

### Ethics Information

Lizards were captured by noosing, and a small (ca 5mm) part of the tail was removed by inducing tail release with a pair of tweezers or, when the tail was regrown, using surgical scissors to provide tissue for genetic analysis. All lizards were released at the site of capture following sampling. The research was approved by the UK Home Office Ethical License PPL30/56 and all work and procedures during fieldwork were carried out under annual licenses and permits from the States of Jersey Government (Department of the Environment) and the French Government (Direction Régionale de l’Environnement, de l’Aménagement et du Logement).

### DNA extraction, sequencing and genotyping

We extracted genomic DNA from tail tissue preserved in ethanol (70–90%) with DNeasy 96 plate kit (Qiagen, Valencia, CA) following manufacturer’s instructions (with overnight lysis). For the phylogenetic analysis we amplified a 656bp region of mitochondrion cytochrome b gene by polymerase chain reaction (PCR) using the primer pair LGlulk [5′-AACCGCCTGTTGTCTTCAACTA-3′] and Hpod [3′-GGTGGAATGGGATTTTGTCTG-5′] [12,26–28]. Amplifications were carried out in a total volume of 15μl consisting of 7.5μl of MyTaq HS Mix (Bioline), 0.45μl (8pm) of each primer (Eurofins), 4.6μl PCR grade H_2_O and 2μl template DNA. PCR conditions were as follows: an initial denaturation step at 94^o^C for 1 min, followed by 35 cycles at 94^o^C for 1 min, 53^o^C for 45sec and 72^o^C for 1 min and a final extension step at 72^o^C for 10min. PCR products were purified using the MinElute 96 UF PCR Purification Kit (Qiagen, Valencia, CA).

Sequencing reactions were carried out with BIGDye Terminator v3.1 Ready Reaction kit (Applied Biosystems, Warrington, UK) in both directions. Products were precipitated in isopropanol and analysed on an ABI 3130 automated capillary sequencer (Applied Biosystems, Warrington, UK). Mitochondrial DNA sequences from both directions were corrected by eye and aligned to obtain a consensus sequence. Accepted sequences were then aligned using MAFFT [29] implemented in Geneious 6.1.7 [30] and trimmed into a uniform length of 656 base pairs (bp). We translated the sequenced *cyt-b* region to amino acid sequences, to verify that no premature stop codons disrupted the reading frame. Unique sequences were submitted to GenBank under the accession numbers KP118978-KP118990.

To infer the genetic structure and diversity of our populations we genotyped 484 individuals at 10 polymorphic microsatellite loci; four described by Richard *et al*. [31] and six recently developed by Heathcote *et al*.[32] (Table A in S1 File). Multiplexed PCRs were carried out in a total volume of 11μl reaction mix containing 1μl of genomic DNA, 5μl of Qiagen MasterMix, 0.2μl of each primer (forward and reverse in equal concentrations) and 3.8μl (for multiplex 1 and 2) or 3.6μl (for multiplex 3) of PCR grade dH_2_O. PCR conditions were as follows: 15min of initialization step at 95^o^C, 26 cycles of 30sec at 94 ^o^C, 90sec at 57 ^o^C (for multiplex 1 and 2) or 55 ^o^C (for multiplex 3) and 1min at 72^o^C and a final extension step of 20min at 60^o^C. The 5’-end of each forward primer was labeled with a fluorescent dye either 6-FAM, HEX or NED. PCR products were run with an internal ladder (red ROX-500), on an ABI 3130 genetic analyser (Applied Biosystems Inc.) We scored alleles in Geneious 6.1.7 and any ambiguous peaks were repeated to confirm genotype.

### Phylogenetic analyses

We used the phylogenetic tree approach to assign haplotypes to known lineages by combining our sequences with 68 sequences (of varying lengths), obtained from GenBank, across the native distribution of the species (see Table B in S1 File [13,25,26,33–36]). Three sequences belonging to *P*. *siculus* (AY185095) [37], *P*. *liolepis* (JQ403296) [38] and *P*. *melisellensis* (AY185097) [37] were used as outgroups in the phylogenetic analysis using Bayesian Inference (BI). We implemented BI analyses in MrBayes [39] under the GTR+G+I nucleotide substitution model as selected by the best-fit model applying the Akaike Information criterion (AIC) in Mega 5.2 [ 40 ]. The BI analysis was run with four chains of 1,000,000 generations and sampling every 100 trees. We discarded (burn-in-length) the first 10% of the trees after checking for convergence of the chains and the posterior probability branch support was estimated from the 50% majority-rule consensus tree.

To investigate evolutionary relationships of our sequences, we constructed a parsimonious phylogenetic network using a median—joining algorithm in Network v.4.6.12 [41]. The method uses median vectors as a hypothetical ancestral sequence required to connect existing sequences within the network with maximum parsimony.

### Population genetics analyses

We checked the microsatellite data in Microchecker V.2.2.3 [42] for null-alleles, large allele dropouts and scoring errors. Basic genetic diversity indices, observed and expected heterozygosities (H_O_, H_E_) were calculated with Genalex 6.5 [43] and allelic richness (A_R_) with Fstat v.2.9.3 [44,45]. Inbreeding coefficient (F_IS_) and deviations from Hardy-Weinberg equilibrium were also evaluated at the 0.05 nominal level for multiple tests using sequential Bonferroni corrections in Fstat v.2.9.3 [44,45]. We compared H_O_, H_E_, A_R_ in island versus mainland populations with a Welch Two Sample t-test and evaluated the correlation between expected heterozygosity and latitude with a Spearman’s rank correlation test in R [46].

To infer population structure, we implemented a Bayesian analysis in Structure v.2.3.4 [47] using the admixture model [48]. The simulations were run with a burn-in of 100,000 iterations and a run length of 10^6^ iterations from K = 1 through 5. Runs for each K were replicated 10 times and the true K was determined according to the method described by Evanno *et al* [49] in the online software Structure Harvester v.0.6.93 [50]. We tested the level of genetic diversity within populations, among populations and among groups (as defined by the structure clustering analysis) by hierarchical analysis of molecular variance (AMOVA, [51]) in Arlequin 3.5.1.3 [52]. Population differentiation was assessed by calculating the F_ST_ values and visualized with a Principle Coordinate Analysis (PCoA) in Genalex 6.5 [43].

## Results

### Phylogeography

**Fig 2:**
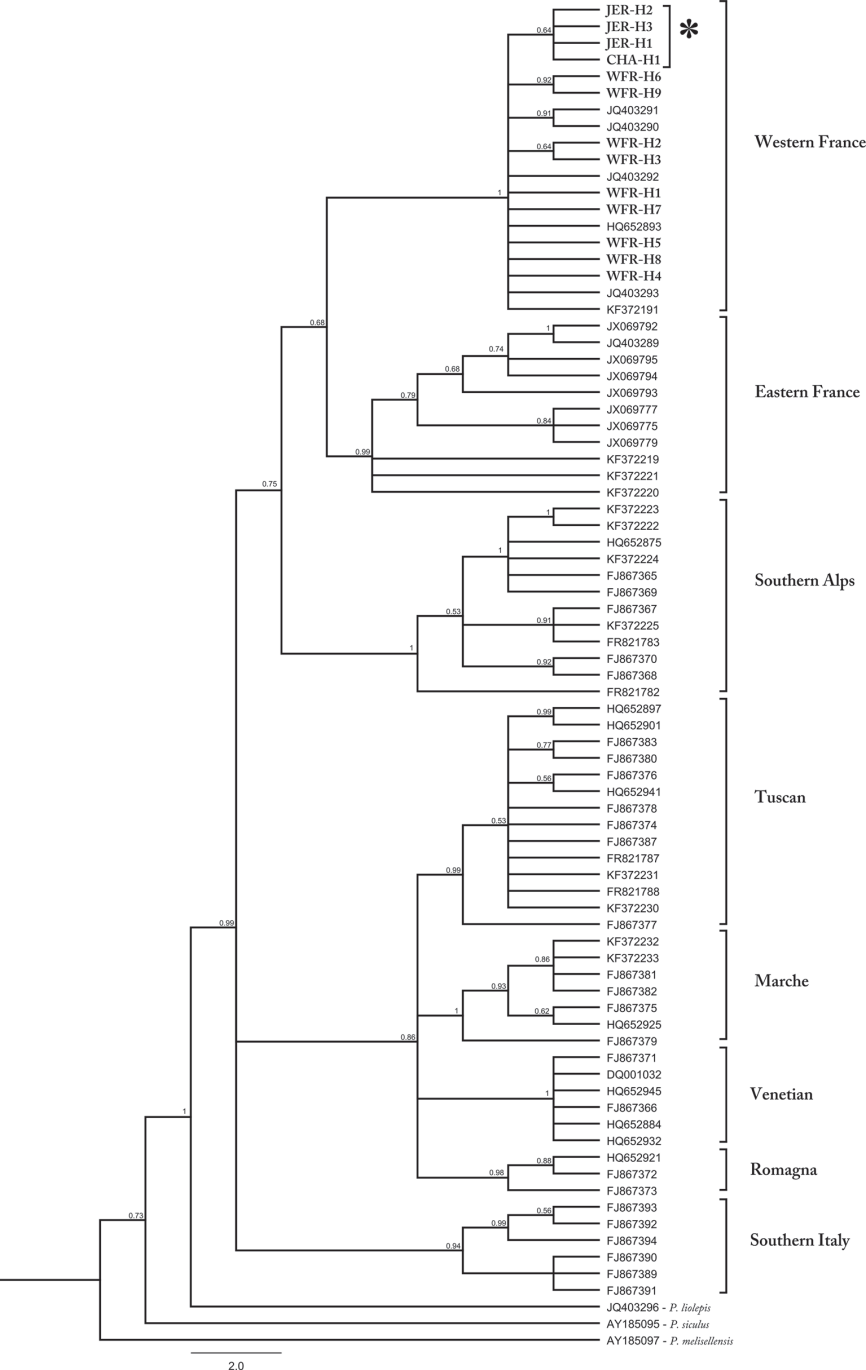
Bayesian inference consensus tree derived from mitochondrial cyt-b sequences. Posterior probabilities (>0.5) are indicated above nodes. Haplotypes analyzed in this study are shown in bold and all were assigned to the Western France Clade. Haplotypes from Jersey and Chausey islands are indicated with an asterisk (*). For information on locality of the sequences see Table B in S1 File.

Analysis of mtDNA sequences of 192 individuals revealed 13 unique haplotypes all nested within the Western France Clade (Fig. 2). The most common haplotype on the mainland (France) was WFR-H5, which was also present on Chausey (one individual) but not on Jersey (Fig. 1A). The parsimony network showed that WFR-H5 has a central position among French haplotypes and JER-H3 forms the centre of the cluster of Jersey and Chausey haplotypes, which are distinct from the rest of the mainland populations (Fig. 1B).

### Population genetics

**Fig 3:**
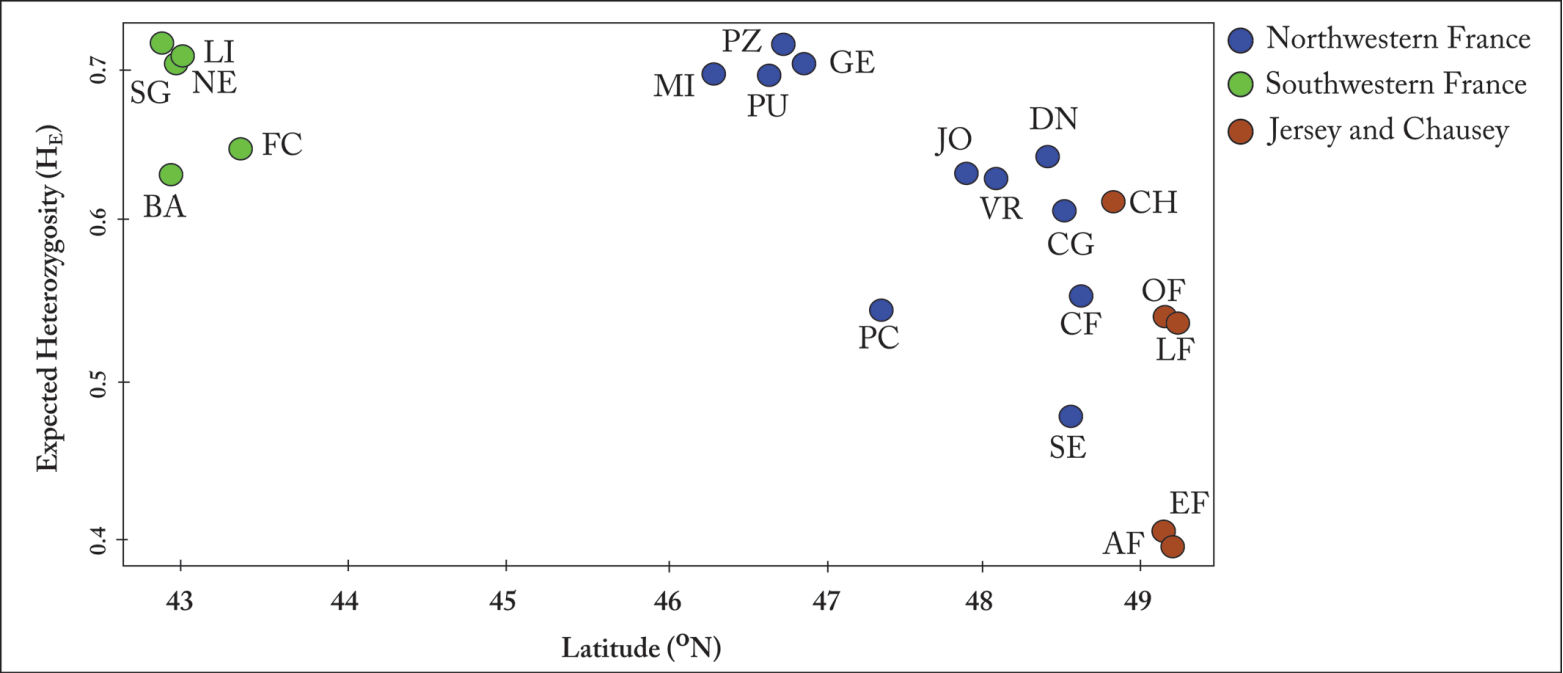
Correlation between expected heterozygosity (H_E_) and latitude. There was a significant negative correlation (r = -0.84, *p <0*.*05*).

All 484 individuals were genotyped at 10 polymorphic loci, ranging from 10 to 56 alleles with mean number of 20.3 alleles per locus across all populations. Evidence of null alleles was observed in several loci but none were consistent across all populations, therefore we did not exclude them for further analysis (Table D in S1 File). Allelic richness, expected and observed heterozygosities (Table 1) were all significantly lower (*p<0*.*05*) in the island populations of Jersey and Chausey than in mainland France populations (Figure B in S1 File). There was a significant negative correlation (r = -0.84, *p <0*.*05*) between latitude and expected heterozygosity (Fig. 3).

**Fig 4:**
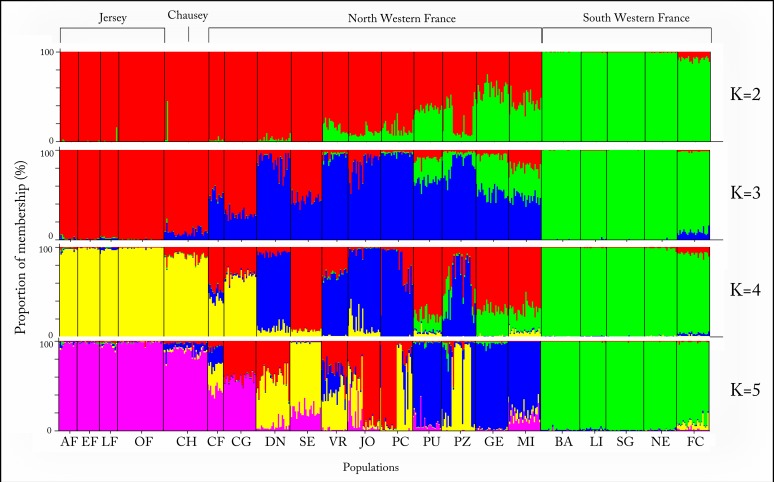
Structure analysis (*K* = 2 to *K* = 5) for all individuals (n = 484). Each individual is represented by a vertical line partitioned into K coloured segments according to the proportion of membership (%) in each cluster. For population abbreviations see Table 1.

**Fig 5:**
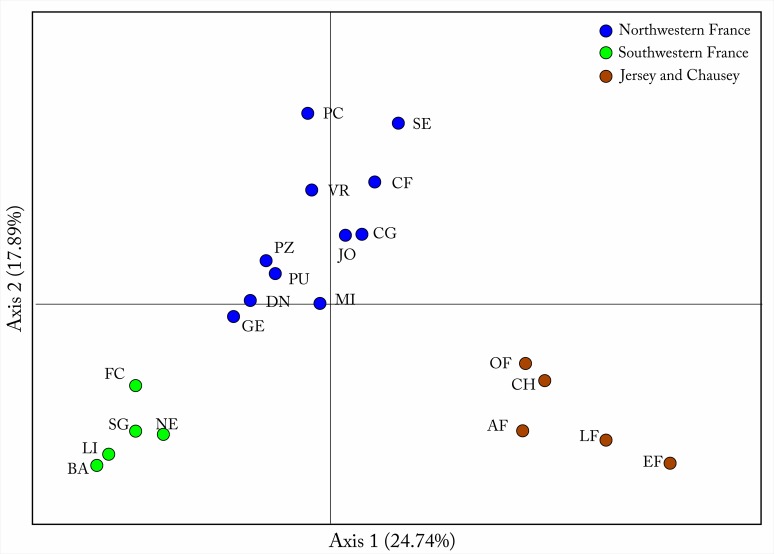
Principle Coordinate Analysis (PCoA) based on F_ST_ values. Three population groups can be identified; the island populations on Jersey and Chausey (bottom right), the north-western French populations (top cluster) and the south-western populations (bottom left).

The Bayesian clustering approach implemented in STRUCTURE suggested *K* = 3 best-fit the genetic data (Fig. 4, see also Figure A in S1 File). The Principle Coordinate Analysis (PCoA) based on F_ST_ values (see Table E in S1 File) between populations confirmed the results from STRUCTURE, identifying three clear groups corresponding to the samples from the Islands, North Western France and South Western France (Fig. 5). Analysis of Molecular Variance (AMOVA) revealed that 28% of the genetic variation was found among the three groups (clusters) and 50% was found within individuals (Table 2).

**Table 2:** Hierarchical analysis of molecular variance (AMOVA)

Source of Variation	d.f	Sum of squares	Variance components	Percentage of variation	Fixation indices	*p* value
**Among groups**	2	686959.219	11893.80493	27.8	F_IS_ = 0.21047	<0.05
**Among populations within groups**	18	342716.546	346.10698	8.09	F_SC_ = 0.11199	<0.05
**Among individuals within populations**	460	1528192.078	577.644	13.5	F_CT_ = 0.27796	<0.05
**Within individuals**	481	1042263.5	2166.86798	50.62	F_IT_ = 0.49377	<0.05
**Total**	961	3600131.344	4280.42425			

## Discussion

Our data provides strong evidence that the wall lizard populations on the islands in the English Channel belong to a single origin. Furthermore, the analyses suggest that this mtDNA clade has been isolated from the mainland for a long period of time and should be considered native. The most parsimonious explanation for the origin of the common wall lizard on Jersey and Chausey Islands appears to be that the increasing sea levels 7000 BP isolated island populations from the mainland and from each other, resulting in independent population histories and hence divergence. It remains possible, however, that there is occasional gene flow between islands. For example, the presence of lizards on very small islets in the Chausey archipelago [18], which are unlikely to be large enough to sustain populations for thousands of years, might indicate that dispersal occasionally occurs between islands. In addition, the presence of the WFR-H5 haplotype on the island of Chausey, which is the most common haplotype on the mainland, might also provide evidence of occasional gene flow between mainland France and the islands. However, it could also be explained by retention of ancestral genetic variation or a more recent introduction. It is worth noting that a single isolated population on the coast of mainland France (Cap Frehel, CF; Fig. 1) also exhibits unique haplotypes, nevertheless it clusters with other mainland populations in all analyses.

Anecdotal evidence suggested that human mediated dispersal might be the most likely explanation for one of the four current locations in Jersey, the population on St. Aubin Fort [23]. Although our mtDNA data revealed a different haplotype from other Jersey populations, the nucDNA clusters all Jersey populations together. This suggests that the source population was most likely animals from other Jersey populations and that the difference in haplotype represents a founder effect.

Overall, these results confirm the suspected native status of Jersey and Chausey wall lizards. Thus, the lower genetic diversity of island populations compared to the mainland populations is expected given the lack of gene flow. This might have significant implication for the long-term persistence of the species on Jersey and Chausey Islands. However, since our data suggests that the species have been present on the islands for thousands of years it might have already been subject to a severe bottleneck that purged deleterious recessives [53]. The species might also have undergone a substantial reduction in abundance more recently. Historical references to the species on Chausey, dated in 1842 [54], and subsequently work recorded the species as very common [55–58]. Despite this, the current distribution of the species on Jersey is very restricted [23]. One partial explanation for this is that lizards on Jersey were part of a wider pet trade, with lizards being sent from Jersey to England as far back as 1761[21]. Indeed, by 1947 the pet trade in lizards had reached such proportions that the local government (States of Jersey) passed the Wildlife Protection (Jersey) Law 1947, which prohibited the buying, selling, exportation or killing of all reptiles and amphibians of Jersey, as a measure to control the increased trade for these animals as pets destined for England (however, none of the contemporary non-native populations in England originate from Jersey [12]). Not only might this explain the current patchy distribution of lizards on Jersey, it might also have contributed to their relatively low genetic variation.

Geographically peripheral populations are often representatives of relatively widespread species within different political boundaries [59]. Their conservation value depends upon their genetic divergence from other conspecific populations because of the synergetic effects of isolation, genetic drift, and natural selection. Whether these range-edge populations merit the conservation effort that they are often subject to has been widely debated [6,60,61]. As this study clarified the native status of the wall lizard population on Jersey, it validates its current full protection status under the Conservation of Wildlife (Jersey) Law 2000 (as amended). The law prohibits the unlicensed taking, sale, keeping, injury and destruction of places for shelter (e.g. nest, dens or burrows) and disturbance of any resident animals. Given our results, it is important that Jersey conservation planners recognize the wall lizard’s restricted distribution, vulnerability to future inbreeding depression, susceptibility to disease, predation and the island’s ever-increasing urban development when developing species management strategies. For instance, should the granite walls and ramparts of historic fortresses where they are in highest abundance be developed or destroyed, the population’s continued survival could be placed at risk. The lizard’s long-term conservation status will depend upon increasing habitat connectivity, especially via coastline protection to connect their north-eastern and eastern coast populations on the island.

## Supporting Information

S1 File Table A, Details for the ten loci used in the study. Multiplexes one (1) and two (2) were developed by Heathcote *et al*. (2014) and multiplex three (3) was developed by Richard *et al*. (2012). Table B, List of sequence data used in the phylogenetic analysis. Information on sampling location, GenBank accession numbers and the reference study. Table C, Historical information on the island populations of the wall lizard. Table D, Table of null alleles per population per locus. Bold values indicated significant deviation from Hardy-Weinberg equilibrium (p<0.05). Table E, Matrix of pairwise *F*_*ST*_ values. Figure A, Plot of Delta K (ΔΚ). Calculated as in Evanno *et al*. (2005) from K = 2 to K = 4. Highest Delta K for *K* = 3. Figure B, Plots of genetic diversity indexes between island (group 1) and mainland populations (group 2). Genetic diversity is expressed as H_O_, H_E_ and A_R_. Differences in the mean numbers were compared with a Welch Two Sample t-test.
(DOCX)
