# Supplementary material for: Phylogeography and Conservation Genetics of the Common Wall Lizard, Podarcis muralis, on Islands at Its Northern Range
Source: PLoS One. 2015 Feb 6;10(2):e0117113. doi: 10.1371/journal.pone.0117113 (PMC4319766; doi:10.1371/journal.pone.0117113)
Supplement: S1 File — Multiplexes one (1) and two (2) were developed by Heathcote et al. (2014) and multiplex three (3) was developed by Richard et al. (2012). Table B, List of sequence data used in the phylogenetic analysis. Information on sampling location, GenBank accession numbers and the reference study. Table C, Historical information on the island populations of the wall lizard. Table D, Table of null alleles per population per locus. Bold values indicated significant deviation from Hardy-Weinberg equilibrium (p<0.05). Table E, Matrix of pairwise F ST values. Figure A, Plot of Delta K (ΔΚ). Calculated as in Evanno et al. (2005) from K = 2 to K = 4. Highest Delta K for K = 3. Figure B, Plots of genetic diversity indexes between island (group 1) and mainland populations (group 2). Genetic diversity is expressed as HO, HE and AR. Differences in the mean numbers were compared with a Welch Two Sample t-test. (DOCX) [file pone.0117113.s001.docx]

Phylogeography and conservation genetics of the common wall lizard, *Podarcis muralis*, on islands at its northern range

Sozos Michaelides^*1§^, Nina Cornish^*2^, Richard Griffiths^3^, Jim Groombridge^3^, Natalia Zajac^1^, Graham J. Walters^4^, Fabien Aubret^5^, Geoffrey M. While^1,6^ & Tobias Uller^1,7§^

^1^Edward Grey Institute, Department of Zoology, University of Oxford, OX1 3PS, Oxford, UK

^2^States of Jersey, Department of the Environment, Howard Davis Farm, La Route de la Trinite, Trinity, Jersey, JE3 5JP, Channel Islands, UK

^3^Durrell Institute of Conservation and Ecology (DICE), School of Anthropology and Conservation, University of Kent, Canterbury, Kent, CT2 7NR, UK

^4^International Institute for Culture, Tourism and Development, London Metropolitan University, 277-281, Holloway Road, London N7 8HN, UK

^5^Station d’Ecologie Expérimentale du CNRS, 09200 Saint-Girons, France

^6^School of Biological Sciences, University of Tasmania, PO Box 55, Hobart, Tas. 7001, Australia

^7^Department of Biology, Lund University, Sölvegatan 37, SE 223 62 Lund, Sweden

^*^ Joint First Authors

^§^ Corresponding authors; emails: [sozos.michaelides@zoo.ox.ac.uk](mailto:sozos.michaelides@zoo.ox.ac.uk); tobias.uller@zoo.ox.ac.uk

**SUPPORTING INFORMATION**

**Table A.** Details for the ten loci used in the study. Multiplexes 1 and 2 were developed by Heathcote *et al* (2014) and multiplex 3 was developed by Richard *et al* (2012).

| **Multiplex** | **Locus** | **Primer sequences** | | **Ta*** | **Product size (bp)** | **Repeat motif** | **Range (bp)** |
| --- | --- | --- | --- | --- | --- | --- | --- |
| 1 | C150 | F | **[6-FAM]**GTCAGCTTTGCAGCACCTTAG | 57 ^o^C | 193 | Di | 175-217 |
|  |  | R | GCGATTAGAGAAGGCGTTTG |  |  |  |  |
|  | C168 | F | **[HEX]**GGTCCGGCTTCAAAGAATAAG |  | 244 | Tetra | 219-350 |
|  |  | R | CAGAGGACTCGCTCAAGGAC |  |  |  |  |
|  | C275-278 | F | **[6-FAM]**GCTTAAAATTAATGCTGCTGCTATTGTATC |  | 245 | Tetra | 210-471 |
|  |  | R | ATAGGTAGAAAATTTATAAACCCTTGG |  |  |  |  |
| 2 | C164 | F | **[6-FAM]**ATCGATGAATGAATGAAGGGCAGT |  | 216 | Tetra | 170-246 |
|  |  | R | CCAGGCATTGTCAAACTATCTG |  |  |  |  |
|  | C038 | F | **[HEX]**CAATGTGCAGTGTTGGGTTG |  | 210 | Tetra | 193-417 |
|  |  | R | ATGTGAGCGACTCCTGGATG |  |  |  |  |
|  | C028 | F | **[6-FAM]**TTGCTTCTGATACGCCTAGC |  | 287 | Tetra | 253-413 |
|  |  | R | AGTGTATTGCGACTGTCAATGG |  |  |  |  |
|  |  |  |  | 55 ^o^C |  |  |  |
| 3 | Pm01 | F | **[6-FAM]** CCACAGGCATCTGGTTAG |  | 128 | Tri | 119-146 |
|  |  |  |  |  |  |  |  |
|  |  | R | TCCATAAGACTGTAAGACAAGCC |  |  |  |  |
|  |  |  |  |  |  |  |  |
|  | Pm05 | F | **[HEX]** CAAGAGGGCAGCCTAGTAATG |  | 160 | Tetra | 135-267 |
|  |  |  |  |  |  |  |  |
|  |  | R | AGATGGGCTCATTTCAACTCC |  |  |  |  |
|  | Pm09 | F | **[NED]** ACGTGTTTCTGTGCTTTGC |  | 189 | Tri | 176-220 |
|  |  | R | AGTCAGACGAGAGGTTGCC |  |  |  |  |
|  | Pm16 | F | **[6-FAM]** GGGATGGAGAAAGATGGCG |  | 192 | Tetra | 179-217 |
|  |  | R | GCACTTGCCTACTGGTCATAC |  |  |  |  |

* Annealing temperature

**Table B. List of sequence data used in the phylogenetic analysis**. Information on sampling location, GenBank accession numbers and the reference study.

| **GenBank accession no.** | **Haplotype code** | **Region – locality – population ID** | **Reference** |
| --- | --- | --- | --- |
| KP118978 | WFR-H7 | Josselin (JO) | This study |
| KP118979 | JER-H1 | Jersey (EF) | This study |
| KP118980 | JER-H3 | Jersey (EF, LF, OF),  Iles de Chausey (GRI, ANE,LGC,IAO, RIR) | This study |
| KP118981 | JER-H2 | Jersey (AF) | This study |
| KP118982 | CHA-H1 | Iles de Chausey (GRE) | This study |
| KP118983 | WFR-H4 | Nebias (NE), Frontier Cabardes (FC) | This study |
| KP118984 | WFR-H5 | Iles de Chausey (GRE), Chateau du Guildo (CG), Dinan (DN), Sees (SE), Vitre (VR), Josselin (JO), Pontchateau (PC), Puybelliard (PU), Pouzagues(PZ), St. Gervais (GE), St. Michel (MI), St. Lizier (LI), St. Girons (SG), Frontier Cabardes (FC) | This study |
| KP118985 | WFR-H9 | Cap Frehel (CF) | This study |
| KP118986 | WFR-H8 | Bastide (BA) | This study |
| KP118987 | WFR-H1 | Cap Frehel (CF) | This study |
| KP118988 | WFR-H2 | St. Lizier (LI), Nebias (NE), Frontiers Cabardes (FC) | This study |
| KP118989 | WFR-H3 | St. Girons (SG), Bastide (BA) | This study |
| KP118990 | WFR-H6 | Cap Frehel (CF) | This study |
| JQ403290 | MS3 | France – Montsegur | Schulte et al. 2012 (Amphibia-Reptilia) |
| JQ403291 | LS6 | France – Lourdes | Schulte et al. 2012 (Amphibia-Reptilia) |
| JQ403292 | LRo1 | France – La Rochelle | Schulte et al. 2012 (Amphibia-Reptilia) |
| JQ403293 | StM1 | France – St Malo | Schulte et al. 2012 (Amphibia-Reptilia) |
| HQ652875 | UU91 | Germany – Bielefeld (previously assigned to the Southern Alps lineage) | Schulte et al. 2008 (Z. Feldherptol),  Schulte et al. 2012 (Global Ecol. Biogeogr) |
| HQ652884 | UU30 | Germany – Dresden (previously assigned to the Venetian lineage) | Schulte et al. 2008 (Z. Feldherptol),  Schulte et al. 2012 (Global Ecol. Biogeogr) |
| HQ652893 | UU67 | Germany – Mainz previously assigned to the Western France lineage) | Schulte et al. 2008 (Z. Feldherptol),  Schulte et al. 2012 (Global Ecol. Biogeogr) |
| HQ652897 | UU76 | Germany – Bad Cannstadt (previously assigned to the Tuscan lineage) | Schulte et al. 2008 (Z. Feldherptol),  Schulte et al. 2012 (Global Ecol. Biogeogr) |
| HQ652901 | UU80 | Germany – Stuttgart (previously assigned to the Tuscany lineage) | Schulte et al. 2008 (Z. Feldherptol),  Schulte et al. 2012 (Global Ecol. Biogeogr) |
| HQ652921 | UU128 | Germany – Lorrach (previously assigned to the Romagna lineage) | Schulte et al. 2008 (Z. Feldherptol),  Schulte et al. 2012 (Global Ecol. Biogeogr) |
| HQ652932 | UU59 | Germany – Aschaffenburg (previously assigned to the Venetian lineage) | Schulte et al. 2008 (Z. Feldherptol),  Schulte et al. 2012 (Global Ecol. Biogeogr) |
| HQ652941 | SD5 | Austria (previously assigned to the Tuscany lineage) | Schulte et al. 2008 (Z. Feldherptol),  Schulte et al. 2012 (Global Ecol. Biogeogr) |
| HQ652945 | UU57 | Germany (previously assigned to the Venetian lineage) | Schulte et al. 2008 (Z. Feldherptol),  Schulte et al. 2012 (Global Ecol. Biogeogr) |
| KF372191 | DB16840 | France – Vielle -Roche | Salvi et al. 2013 (BMC Evol. Bio) |
| KF372219 | DB13461 | France – Massif des Maures | Salvi et al. 2013 (BMC Evol. Bio) |
| KF372220 | DB13460 | France – Valle de Gilly | Salvi et al. 2013 (BMC Evol. Bio) |
| KF372221 | DB13430 | France – Massif des Maures | Salvi et al. 2013 (BMC Evol. Bio) |
| KF372222 | DBM3 | Italy – Viozene | Salvi et al. 2013 (BMC Evol. Bio) |
| KF372223 | DBM1 | Italy – Viozene | Salvi et al. 2013 (BMC Evol. Bio) |
| KF372224 | DB15936 | Switzerland – Monte Verita | Salvi et al. 2013 (BMC Evol. Bio) |
| KF372225 | DB16837 | Italy – Bianzano | Salvi et al. 2013 (BMC Evol. Bio) |
| KF372230 | DB1399 | Italy – Ostia Antica | Salvi et al. 2013 (BMC Evol. Bio) |
| KF372231 | DB5938 | Italy – Paganico | Salvi et al. 2013 (BMC Evol. Bio) |
| KF372232 | DMM39 | Italy – Majelletta | Salvi et al. 2013 (BMC Evol. Bio) |
| KF372233 | DMM40 | Italy – Majelletta | Salvi et al. 2013 (BMC Evol. Bio) |
| DQ001032 | mur12 | Italy – Friuli-Venezia | Podnar et al. 2007 (J. Mol. Evol) |
| FJ867365 | H1 | Italy – Trento, Vercelli | Giovannotti et al. 2010 (Ital. J. Zool.) |
| FJ867366 | H2 | Italy – Trieste | Giovannotti et al. 2010 (Ital. J. Zool.) |
| FJ867367 | H3 | Italy – Pavia | Giovannotti et al. 2010 (Ital. J. Zool.) |
| FJ867368 | H4 | Italy – Pavia | Giovannotti et al. 2010 (Ital. J. Zool.) |
| FJ867369 | H5 | Italy - Val Germanasca | Giovannotti et al. 2010 (Ital. J. Zool.) |
| FJ867370 | H6 | Italy – Parma | Giovannotti et al. 2010 (Ital. J. Zool.) |
| FJ867371 | H7 | Italy – Ferrara, Ravenna | Giovannotti et al. 2010 (Ital. J. Zool.) |
| FJ867372 | H8 | Italy – Ravenna, Cesena | Giovannotti et al. 2010 (Ital. J. Zool.) |
| FJ867373 | H9 | Italy – Cesena, Pesaro, Montignano, Senigallia | Giovannotti et al. 2010 (Ital. J. Zool.) |
| FJ867374 | H10 | Italy – Carpegna, Bolognola, Amatrice, Gran Sasso, L’Aquila, Latina | Giovannotti et al. 2010 (Ital. J. Zool.) |
| FJ867375 | H11 | Italy – Montifnano, Ancona | Giovannotti et al. 2010 (Ital. J. Zool.) |
| FJ867376 | H12 | Italy – Pisa | Giovannotti et al. 2010 (Ital. J. Zool.) |
| FJ867377 | H13 | Italy – Genga, M. te San Vicino | Giovannotti et al. 2010 (Ital. J. Zool.) |
| FJ867378 | H14 | Italy – Genga | Giovannotti et al. 2010 (Ital. J. Zool.) |
| FJ867379 | H15 | Italy – M. te San Vicino, Porto S. Elpidio, Macerata, Bolognola, Porto d’ Ascoli | Giovannotti et al. 2010 (Ital. J. Zool.) |
| FJ867380 | H16 | Italy – Viso | Giovannotti et al. 2010 (Ital. J. Zool.) |
| FJ867381 | H17 | Italy – Caramanico Terme | Giovannotti et al. 2010 (Ital. J. Zool.) |
| FJ867382 | H18 | Italy – Gran Sasso | Giovannotti et al. 2010 (Ital. J. Zool.) |
| FJ867383 | H19 | Italy – L’ Aquila | Giovannotti et al. 2010 (Ital. J. Zool.) |
| FJ867389 | H25 | Italy – Monti Alburni | Giovannotti et al. 2010 (Ital. J. Zool.) |
| FJ867390 | H26 | Italy – Monti Alburni | Giovannotti et al. 2010 (Ital. J. Zool.) |
| FJ867391 | H27 | Italy – Monti Alburni | Giovannotti et al. 2010 (Ital. J. Zool.) |
| FJ867392 | H28 | Italy – Pollino | Giovannotti et al. 2010 (Ital. J. Zool.) |
| FJ867393 | H29 | Italy – Pollino | Giovannotti et al. 2010 (Ital. J. Zool.) |
| FJ867394 | H30 | Italy – Pollino | Giovannotti et al. 2010 (Ital. J. Zool.) |
| JX069775 | Mont St Odily | France – Mont St Odily | Gassert et al. 2013 (J. Biogeography) |
| JX069777 | Bitche | France – Bitche | Gassert et al. 2013 (J. Biogeography) |
| JX069779 | Euville | France – Euville | Gassert et al. 2013 (J. Biogeography) |
| JX069792 | Labeaume | France – Labeaume | Gassert et al. 2013 (J. Biogeography) |
| JX069793 | St Remy | France – Saint Remy de Provence | Gassert et al. 2013 (J. Biogeography) |
| JX069794 | Autun | France – Autun | Gassert et al. 2013 (J. Biogeography) |
| JX069795 | St Martin | France – Saint Martin | Gassert et al. 2013 (J. Biogeography) |
| FR821782 | M2 | Italy – Pavia | Bellati et al. 2011 (J. Zool. Syst. Evol. Res) |
| FR821783 | M3 | Italy – Bereguardo | Bellati et al. 2011 (J. Zool. Syst. Evol. Res) |
| FR821787 | N3 | Italy – Borgo Montello | Bellati et al. 2011 (J. Zool. Syst. Evol. Res) |
| FR821788 | N4 | Italy – Borgo Montello | Bellati et al. 2011 (J. Zool. Syst. Evol. Res) |
| AY185095 | *P. siculus* | Outgroup | Podnar et al. 2004 (Organisms Div. Evol) |
| AY185097 | *P. melisellensis* | Outgroup | Podnar et al. 2004 (Organisms Div. Evol) |
| JQ403296 | *P. liolepis* | Outgroup | Schulte et al. 2012 (Amphibia-Reptilia) |

**Table C.** Historical information on the island populations of the wall lizard.

| **Sampled Location** | **Information** |
| --- | --- |
| St. Aubin Fort (AF) | The fort is located in St Aubin's Bay on the southast coastline of Jersey and dates back to the 16th century (1540s). The population of wall lizard is thought to be a relatively recent introduction (Le Sueur, 1976). There are some records from site managers and gardeners that the lizards have been abundant on the fort since the 1940s. Since then, other sites along the south coast of Jersey have been identified, in both private and public gardens. Smith, (2000) estimated the total minimum number of lizards at St Aubin’s Fort to be 54 ± 3.80 individuals. |
| Fort Leicester  (LF) | The fort is built into a hillside above Bouley Bay on Jersey’s northeastern coastline. It was constructed in 1836 as part of an island-wide defense strategy against French invasion. Subsequently the Fort has undergone numerous modifications made by the occupying forces during the Second World War (Hills, 2005a at the end of a small pier used for fisheries and coastal defense. The average number of lizards at the Fort was calculated to be a minimum of 70 ±17.39 individuals (Smith, 2000). |
| L’Etacquerel Fort  (EF) | L’Etacquerel Fort, essentially a gun battery, was developed as part of a late 18th to early 19^th^ century island-wide defense strategy (Hills, 2005b). The Fort is built on a headland on the east side of Fort Leicester, Bouley Bay. The total minimum number of adult lizards calculated was 26 ± 13.83 individuals (Smith, 2000). |
| Mont Orgueil Castle and Gorey  (OF) | Mont Orgueil castle situated on Jersey’s eastern coastline was built in several stages from the 13th century onwards. The construction of the castle was undertaken from 1204 as the main defence for the Island. During the 17th century the castle was no longer the primary defence for the Island so it was regarded as the Islands only prison until the end of 17th century, when the castle was stated as a ruin and subsequently abandoned. Eventually repairs were carried out over the 18th to 19th centuries and the castle was open to the public and has been classed as a museum site since 1929. The castle was also altered during the Second World War by German forces but continued as a museum after the occupation. Mont Orgueil castle has the largest population of *P. muralis* on Jersey which is widely distributed around the castles walls and extensive gardens (Hall, 2003). |
| Chausey Island  (CHA) | Iles Chausey archipelago consists of 52 islands totaling approximately 59ha [[18](#_ENREF_18)]. Thirteen islands have been confirmed the presence of the species. We sampled lizards from Grand Ile (GRI; the largest island, 39h and the largest population), Aneret (ANE), le Grand Colombier (LGC), Grand Epail (GRE), Riche Roche (RIR) and Iles aux Oiseaux (IAO). |

**Table D.** Table of null alleles per population per locus. Bold values indicated significant deviation from Hardy-Weinberg equilibrium (p<0.05).

|  | **AF** | **EF** | **LF** | **OF** | **CH** | **CF** | **CG** | **DN** | **SE** | **VR** | **JO** | **PC** | **PU** | **PZ** | **GE** | **MI** | **BA** | **LI** | **SG** | **NE** | **FC** |
| --- | --- | --- | --- | --- | --- | --- | --- | --- | --- | --- | --- | --- | --- | --- | --- | --- | --- | --- | --- | --- | --- |
| Locus1 | **0.33** | **0.33** | **0.33** | **0.32** | **0.27** | **0.24** | 0.03 | **0.01** | 0.00 | **0.10** | 0.12 | **0.05** | **0.01** | 0.02 | 0.01 | 0.00 | 0.00 | 0.03 | 0.02 | **0.00** | **0.05** |
| Locus2 | 0.17 | 0.30 | 0.35 | **0.25** | 0.00 | 0.00 | **0.18** | **0.04** | **0.23** | **0.00** | 0.02 | **0.10** | 0.10 | 0.06 | **0.09** | **0.00** | 0.33 | 0.07 | **0.42** | **0.24** | **0.20** |
| Locus3 | **0.17** | 0.11 | 0.00 | 0.11 | 0.02 | 0.00 | **0.10** | **0.16** | 0.00 | 0.09 | **0.17** | 0.00 | 0.03 | 0.00 | 0.01 | 0.10 | 0.00 | 0.01 | 0.00 | 0.02 | 0.02 |
| Locus4 | **0.18** | No info | No info | **0.12** | **0.12** | No info | No info | 0.00 | No info | No info | No info | 0.00 | No info | 0.00 | No info | No info | No info | 0.00 | **0.00** | **0.00** | No info |
| Locus5 | **0.25** | 0.00 | 0.07 | **0.10** | 0.01 | 0.02 | 0.20 | 0.00 | 0.00 | 0.05 | 0.00 | 0.00 | 0.00 | 0.00 | 0.13 | 0.04 | 0.00 | 0.00 | 0.15 | 0.05 | 0.10 |
| Locus6 | **0.13** | **0.04** | 0.00 | **0.05** | 0.00 | 0.00 | 0.00 | 0.00 | 0.07 | 0.01 | **0.01** | 0.02 | 0.00 | **0.02** | 0.00 | 0.00 | **0.01** | 0.00 | 0.00 | 0.02 | 0.05 |
| Locus7 | 0.00 | 0.00 | 0.06 | 0.00 | 0.00 | No info | 0.07 | 0.00 | 0.00 | **0.00** | **0.01** | 0.00 | 0.01 | **0.05** | 0.00 | 0.05 | 0.00 | 0.00 | 0.00 | 0.03 | 0.01 |
| Locus8 | 0.00 | **0.10** | 0.04 | 0.00 | 0.00 | 0.16 | 0.00 | 0.08 | 0.00 | 0.05 | 0.00 | 0.00 | 0.08 | 0.03 | 0.09 | 0.00 | 0.05 | 0.00 | 0.00 | 0.03 | **0.00** |
| Locus9 | **0.25** | No info | **0.27** | **0.16** | **0.10** | 0.04 | 0.13 | **0.00** | 0.00 | 0.09 | **0.05** | **0.06** | 0.01 | **0.00** | 0.00 | **0.00** | 0.04 | **0.00** | 0.00 | 0.11 | 0.00 |
| Locus10 | 0.00 | 0.00 | 0.22 | 0.07 | 0.06 | 0.06 | 0.10 | 0.00 | 0.02 | 0.00 | 0.00 | 0.00 | 0.02 | 0.04 | 0.05 | 0.00 | 0.04 | **0.04** | 0.00 | **0.05** | 0.01 |

**Table E.** Matrix of pairwise *F_ST_* values

|  | AF | EF | LF | OF | CH | CF | CG | DN | SE | VR | JO | PC | PU | PZ | GE | MI | BA | LI | SG | NE | FC |
| --- | --- | --- | --- | --- | --- | --- | --- | --- | --- | --- | --- | --- | --- | --- | --- | --- | --- | --- | --- | --- | --- |
| AF | 0.000 |  |  |  |  |  |  |  |  |  |  |  |  |  |  |  |  |  |  |  |  |
| EF | 0.251 | 0.000 |  |  |  |  |  |  |  |  |  |  |  |  |  |  |  |  |  |  |  |
| LF | 0.171 | 0.054 | 0.000 |  |  |  |  |  |  |  |  |  |  |  |  |  |  |  |  |  |  |
| OF | 0.160 | 0.130 | 0.085 | 0.000 |  |  |  |  |  |  |  |  |  |  |  |  |  |  |  |  |  |
| CH | 0.128 | 0.100 | 0.058 | 0.076 | 0.000 |  |  |  |  |  |  |  |  |  |  |  |  |  |  |  |  |
| CF | 0.199 | 0.203 | 0.160 | 0.152 | 0.112 | 0.000 |  |  |  |  |  |  |  |  |  |  |  |  |  |  |  |
| CG | 0.206 | 0.198 | 0.149 | 0.128 | 0.097 | 0.082 | 0.000 |  |  |  |  |  |  |  |  |  |  |  |  |  |  |
| DN | 0.221 | 0.180 | 0.152 | 0.141 | 0.133 | 0.120 | 0.080 | 0.000 |  |  |  |  |  |  |  |  |  |  |  |  |  |
| SE | 0.259 | 0.234 | 0.175 | 0.144 | 0.144 | 0.155 | 0.182 | 0.183 | 0.000 |  |  |  |  |  |  |  |  |  |  |  |  |
| VR | 0.200 | 0.211 | 0.147 | 0.125 | 0.105 | 0.116 | 0.113 | 0.110 | 0.094 | 0.000 |  |  |  |  |  |  |  |  |  |  |  |
| JO | 0.146 | 0.191 | 0.132 | 0.101 | 0.094 | 0.104 | 0.106 | 0.099 | 0.105 | 0.053 | 0.000 |  |  |  |  |  |  |  |  |  |  |
| PC | 0.248 | 0.213 | 0.161 | 0.152 | 0.151 | 0.073 | 0.086 | 0.090 | 0.114 | 0.083 | 0.087 | 0.000 |  |  |  |  |  |  |  |  |  |
| PU | 0.155 | 0.174 | 0.127 | 0.102 | 0.093 | 0.098 | 0.093 | 0.076 | 0.108 | 0.059 | 0.051 | 0.069 | 0.000 |  |  |  |  |  |  |  |  |
| PZ | 0.167 | 0.156 | 0.109 | 0.104 | 0.095 | 0.078 | 0.075 | 0.063 | 0.091 | 0.042 | 0.044 | 0.052 | 0.014 | 0.000 |  |  |  |  |  |  |  |
| GE | 0.171 | 0.181 | 0.130 | 0.108 | 0.099 | 0.113 | 0.101 | 0.084 | 0.121 | 0.073 | 0.062 | 0.091 | 0.022 | 0.020 | 0.000 |  |  |  |  |  |  |
| BA | 0.167 | 0.140 | 0.093 | 0.093 | 0.078 | 0.095 | 0.089 | 0.070 | 0.101 | 0.066 | 0.063 | 0.070 | 0.030 | 0.023 | 0.037 | 0.000 |  |  |  |  |  |
| MI | 0.216 | 0.234 | 0.172 | 0.157 | 0.139 | 0.170 | 0.159 | 0.115 | 0.200 | 0.132 | 0.123 | 0.157 | 0.085 | 0.070 | 0.069 | 0.082 | 0.000 |  |  |  |  |
| LI | 0.222 | 0.213 | 0.161 | 0.160 | 0.138 | 0.154 | 0.135 | 0.092 | 0.197 | 0.122 | 0.120 | 0.152 | 0.078 | 0.065 | 0.066 | 0.077 | 0.039 | 0.000 |  |  |  |
| SG | 0.201 | 0.196 | 0.144 | 0.140 | 0.127 | 0.137 | 0.122 | 0.083 | 0.170 | 0.107 | 0.095 | 0.134 | 0.063 | 0.053 | 0.050 | 0.059 | 0.034 | 0.022 | 0.000 |  |  |
| NE | 0.181 | 0.185 | 0.130 | 0.125 | 0.119 | 0.131 | 0.119 | 0.086 | 0.159 | 0.102 | 0.092 | 0.132 | 0.064 | 0.053 | 0.051 | 0.057 | 0.033 | 0.024 | 0.021 | 0.000 |  |
| FC | 0.199 | 0.214 | 0.156 | 0.133 | 0.126 | 0.127 | 0.121 | 0.085 | 0.165 | 0.102 | 0.090 | 0.113 | 0.065 | 0.050 | 0.055 | 0.069 | 0.040 | 0.043 | 0.036 | 0.030 | 0.000 |

**Figure A**. Plot of Delta K (ΔΚ). Calculated as in Evanno *et al.* (2005) from K 2 to K 4. Highest Delta K for *K*=3.


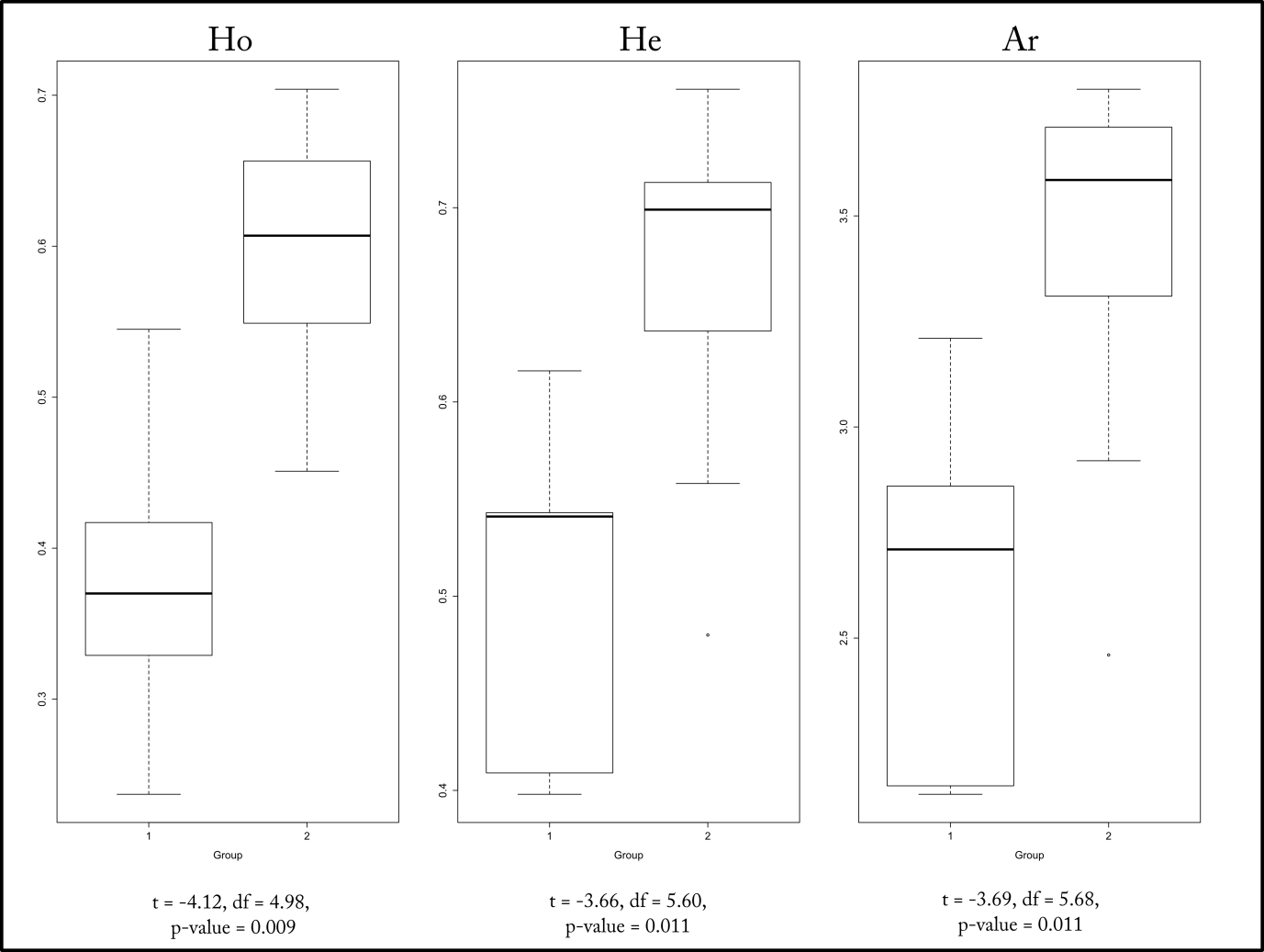


**Figure B.** **Plots of genetic diversity indexes between island (group 1) and mainland populations (group 2).** Genetic diversity is expressed as Ho, He and Ar. Differences in the mean numbers were compared with a Welch Two Sample t-test.

**References**

Earl Da, vonHoldt BM (2011) STRUCTURE HARVESTER: a website and program for visualizing STRUCTURE output and implementing the Evanno method. Conservation Genetics Resources 4: 359-361.

Evanno, G., Regnaut, S. & Goudet, J. (2005) Detecting the number of clusters of individuals using the software STRUCTURE: a simulation study. *Molecular Ecology*, **14**, 2611-20.

Hall, N. C. (2003) Investigation of distribution of the Jersey Wall Lizard *Podarcis muralis* at Mont Orgueil Castle. States of Jersey. Planning and Environment Department. *Unpublished report*.

Heathcote RJP, Dawson DA, Uller T (2014) Characterisation of nine European wall lizard (*Podarcis muralis*) microsatellite loci of utility across sub-species. Conservation Genetics Resources.

Hills, R. (2005a) Fort Leicester Conservation Statement. Jersey Heritage Trust. *Unpublished report*.

Hills, R. (2005b) L’Etacquerel Fort Conservation Statement. Jersey Heritage Trust. *Unpublished report.*

Le Sueur, F. (1976) *A Natural History of Jersey*. Phillimore & Co. Ltd, London & Chichester.

Richard M, Stevens VM, Hénanff ML, Coulon A (2012) Fourteen new polymorphic microsatellite loci for the wall lizard *Podarcis muralis* ( Sauria : Lacertidae ). Molecular Ecology Resources: 1-5.

Smith, R. (2000) Census of Jersey wall lizards *Podarcis muralis* and ecological correlates of distribution at fort sites in Jersey. *Dodo,* 36**,** 95-96.

Walters, G.J. & Ineich, I. (2006) Insular populations of the lizard *Podarcis muralis* at the northwestern limit of its range *Bulletin de la Societe zoologique de France*, **131**
